# Risk of Gastrointestinal Adverse Events in Cancer Patients Treated With Immune Checkpoint Inhibitor Plus Chemotherapy: A Systematic Review and Meta-Analysis

**DOI:** 10.3389/fonc.2020.00197

**Published:** 2020-03-10

**Authors:** Wenhan Yang, Peng Men, Huimin Xue, Mingyan Jiang, Qiuhua Luo

**Affiliations:** ^1^Department of Pharmacy, The First Hospital of China Medical University, Shenyang, China; ^2^School of Pharmacy, China Medical University, Shenyang, China; ^3^Department of Pharmacy, Peking University Third Hospital, Beijing, China

**Keywords:** gastrointestinal adverse events, immune checkpoint inhibitor, programmed death 1, programmed death ligand 1, cytotoxic T-lymphocyte-associated protein 4, chemotherapy

## Abstract

**Background:** The combination of immune checkpoint inhibitors (ICIs) and chemotherapy can improve clinical outcomes in the treatment of various tumors, but may also be associated with more adverse events (AEs). We performed a systematic review and meta-analysis to characterize the risk of gastrointestinal AEs in cancer patients treated with ICI plus chemotherapy.

**Methods:** This review was based on comprehensive search through PubMed, EMBASE, and the Cochrane Library for randomized controlled trials (RCTs) that reported gastrointestinal AEs following the use of ICI plus chemotherapy. Literature screening, data extraction, and quality evaluation were performed by two individual reviewers. Revman (version 5.3) was used for meta-analysis. Risk ratios (RR) with 95% confidence interval (CI) were calculated. Meta-analysis was conducted according to different types of ICIs [programmed death 1 (PD-1), programmed death ligand 1 (PD-L1), and cytotoxic T-lymphocyte-associated protein 4 (CTLA-4) inhibitors].

**Results:** After a full-text review, 10 trials involving 5,142 patients were included in the study. Compared with chemotherapy alone, PD-1 inhibitor plus chemotherapy significantly increased the risk of diarrhea (RR = 1.38, 95% CI, 1.13–1.68, *P* = 0.001; *I*^2^ = 0%) and colitis (RR = 2.90, 95% CI, 1.02–8.21, *P* = 0.050; *I*^2^ = 0%), PD-L1 inhibitor plus chemotherapy significantly increased the risk of nausea (RR = 1.17, 95% CI, 1.02-1.35, *P* = 0.020; *I*^2^ = 0%), while CTLA-4 inhibitor plus chemotherapy significantly increased the risk of decreased appetite (RR = 1.49, 95% CI, 1.17–1.90, *P* = 0.001; *I*^2^ = 0%), diarrhea (RR = 2.23, 95% CI, 1.90–2.63, *P* < 0.00001; *I*^2^ = 0%), and colitis (RR = 28.39, 95% CI, 5.59–144.24, *P* < 0.001; *I*^2^ = 0%).

**Conclusions:** This meta-analysis demonstrated that ICI plus chemotherapy is associated with a higher risk of gastrointestinal AEs. However, combining different ICIs may lead to diverse gastrointestinal toxicities. Clinicians should be aware of these AEs in the application of ICI plus chemotherapy.

## Introduction

Cancer therapy has made great progress with the advance of immune checkpoint inhibitors (ICIs) including cytotoxic T-lymphocyte-associated protein 4 (CTLA-4), programmed death 1 (PD-1), and programmed death ligand 1 (PD-L1) inhibitors. So far, they have been approved for the treatment of melanoma, metastatic renal cell carcinoma (RCC), non–small-cell lung cancer (NSCLC), head and neck cancer, and hematological system diseases (1–7). The ICI therapy brings out lasting therapeutic effect by allowing augmentation of immunologic response against tumor cells; however, it covers only a minority of patients with the objective response rate (ORR) of no more than 30% (8, 9). Many clinical trials have proved that the combination of ICI and chemotherapy could further improve patient response to drugs, duration, and other clinical outcomes (10–17). In January 2019, the US Food and Drug Administration (FDA) accepted atezolizumab plus carboplatin and nab-paclitaxel as the first-line treatment option for metastatic NSCLC patients without epidermal growth factor receptor (EGFR) or anaplastic lymphoma kinase (ALK) mutations. In addition, pembrolizumab combined with pemetrexed and carboplatin have been approved for the first-line treatment of non-squamous NSCLC, regardless of the expression of PD-L1. Furthermore, some phase II/III clinical studies are also making progress involving combination therapy (18, 19).

Despite the effectiveness of ICI plus chemotherapy, it may also be associated with more adverse events (AEs) (20, 21). In ICI therapy, widespread activation of T cells, coupled with the depletion of regulatory T cells, causes an attack on various organ systems and leads to a spectrum of AEs known as immune-related adverse events (irAEs). These irAEs have been commonly found to affect the endocrine, respiratory, musculoskeletal, cardiovascular, hematologic, and gastrointestinal system. Gastrointestinal irAEs range from mild diarrhea to severe colitis and are the most commonly reported grade 3–4 irAEs that might be challenging to treat (22). On the other hand, traditional chemotherapy is also associated with multiple gastrointestinal AEs, such as nausea, decreased appetite, diarrhea, and vomiting, which may greatly reduce the patient-based compliance of medication and even result in treatment termination. Consequently, while achieving clinical benefits, whether there is a superposition of gastrointestinal side effects in the combination therapy remains unknown. Given the increasing application of ICI plus chemotherapy, clinicians have an urgent need for more information around the issue. We performed a meta-analysis of randomized clinical trials (RCTs) to characterize the risk of gastrointestinal AEs associated with ICI plus chemotherapy regimen.

## Methods

### Search Methods and Study Selection

The literature search was performed by two individual reviewers. PubMed, EMBASE, and the Cochrane Library were searched from the inception of the database to March 2019. Keywords were “ipilimumab,” “pembrolizumab,” “nivolumab,” “atezolizumab,” “tremelimumab,” “durvalumab,” “avelumab,” “CTLA-4,” “PD-1,” “PD-L1,” and “immune checkpoint inhibitor.” Reference lists in the retrieved articles and supplemental materials were also examined manually to further identify any potentially relevant trials. The eligibility criteria for the systematic review were in accordance with the PICO (participants, intervention, comparison, and outcomes) approach. Furthermore, the drug manufacturers' websites were searched for any additional information.

RCTs meeting the following criteria were considered for inclusion:

- Participants: patients with malignant tumors.
- Intervention: ICI combined with chemotherapy.
- Comparison: chemotherapy.
- Outcomes: nausea, constipation, diarrhea, decreased appetite, vomiting and colitis.

If more than one publication reported a same study, only the most complete, updated data were included in this analysis. Two reviewers independently screened all titles, abstracts, and full texts for eligibility. Any discrepancy among investigators was resolved by consensus.

### Data Extraction and Quality Assessment

The data extraction was performed by two individual reviewers. The following baseline characteristic items of the included studies were extracted from each study: the year of study, trial phase, treatment regimen, number of patients, median age, sex proportion of patients, and cancer types. The following clinical outcomes data were also extracted: the number of patients treated with ICI plus chemotherapy regimen, the number of patients treated with chemotherapy alone, and the number of patients with each gastrointestinal AE. Any discrepancy among investigators was resolved by consensus. Two reviewers also independently assessed the qualities of the included studies. Discrepancies were resolved by discussion or through consultation with a third reviewer. The potential risk of bias in these RCTs was assessed according to criteria developed with the Cochrane risk-of-bias tool (23).

### Statistical Analysis

Revman (version 5.3) was used for meta-analysis. Risk ratios (RRs) with 95% confidence intervals (CIs) were calculated. The Cochran *Q* statistic and *I*^2^ statistics were used to examine heterogeneity. The random-effect model was chosen if significant heterogeneity existed (*I*^2^ > 50%); otherwise, the fixed-effect model was used. Meta-analysis was conducted according to the type of ICI medications (PD-1, PD-L1, and CTLA-4 inhibitors). The funnel plots were used to examine the publication bias among studies.

## Results

### Literature Search

**Figure 1:**
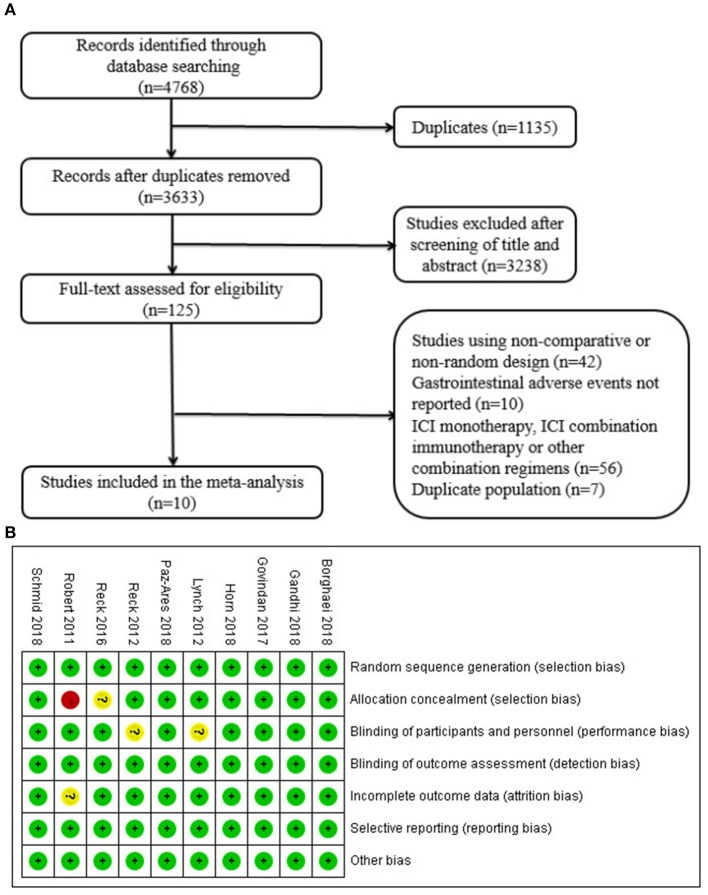
**(A)** Flow diagram of study selection. Database searching was based on PubMed, EMBASE, and the Cochrane Library. **(B)** Quality assessment for 10 included studies. Quality of trials was categorized into three grades: low risk of bias (+), high risk of bias (–), and unclear (?). ICI, immune checkpoint inhibitor.

A total of 4,768 records were identified from the initial search. After screening the titles and abstracts, 125 studies were selected for full-text review. Finally, 10 trials involving 5,142 patients were included in our analysis (Figure 1A). Three studies were phase II trials (12, 15, 16) and seven were phase III trials (10, 11, 13, 14, 17–19). Three trials investigated pembrolizumab plus chemotherapy (10–12), two investigated atezolizumab inhibitor plus chemotherapy (13, 14), and the other five investigated ipilimumab plus inhibitor chemotherapy (15–19). Types of cancer were NSCLC in five trials (*n* = 2198) (10–12, 15, 18), small-cell lung cancer in three trials (*n* = 1,487) (14, 16, 17), triple-negative breast cancer in one trial (*n* = 902) (13), and melanoma in the other trial (*n* = 502) (19). The detailed baseline characteristics are shown in Table 1.

**Table 1:** Characteristics of the included studies.

**Study**	**Year**	**Phase**	**Treatment arms**	**No. of patients**	**Median age (years)**	**Male (%)**	**Cancer type**	**Follow-up time (months)**	**NCT number**
Paz-Ares et al. (10)	2018	3	Pembrolizumab+carboplatin+paclitaxel/nab-paclitaxel	278	65 (29–87)	220 (79.1)	Squamous non–small-cell lung cancer	21	02775435
			Placebo+carboplatin+paclitaxel/nab-paclitaxel	281	65 (36–88)	235 (83.6)			
Gandhi et al. (11)	2018	3	Pembrolizumab+pemetrexed+platinum	410	65 (34–84)	254 (62.0)	Metastatic non–small-cell lung cancer	21	02578680
			Placebo+pemetrexed+platinum	206	63.5 (34–84)	109 (52.9)			
Borghaei et al. (12)	2018	2	Pembrolizumab+pemetrexed+carboplatin	60	62.5 (54–70)	22 (37)	Advanced non-small cell lung cancer	36	02039674
			Pemetrexed+carboplatin	63	63.2 (58–70)	26 (41)			
Schmid et al. (13)	2018	3	Atezolizumab+nab-paclitaxed	451	55 (20–82)	3 (0.7)	Advanced triple-negative breast cancer	36	02425891
			Placebo+nab-paclitaxel	451	56 (26–86)	1 (0.2)			
Horn et al. (14)	2018	3	Atezolizumab+carboplatin+etoposide	201	64 (28–60)	129 (64.2)	Extensive-stage small-cell lung cancer	24	02763579
			Placebo+carboplatin+etoposide	202	64 (26–87)	132 (65.3)			
Lynch et al. (15)	2012	2	Ipilimumab+carboplatin+paclitaxel	138	60 (36–88)	102 (74)	Stage IIIB/IV non–small-cell lung cancer	26	00527735
			Placebo+carboplatin+paclitaxel	66	62 (36–82)	49 (74)			
Reck et al. (16)	2012	2	Ipilimumab+paclitaxel/carboplatin	85	58 (43–80)	65 (76)	Extensive-stage small-cell lung cancer	24	00527735
			Placebo+paclitaxel/carboplatin	45	58 (42–82)	33 (73)			
Reck et al. (17)	2016	3	Ipilimumab+platinum+etoposide	478	62 (39–85)	317 (66)	Extensive-stage small-cell lung cancer	32	01450761
			Placebo+platinum+etoposide	476	63 (36–81)	326 (68)			
Govindan et al. (18)	2017	3	Ipilimumab+paclitaxel+carboplatin	388	64 (28–84)	326 (84)	Advanced squamous non–small-cell lung cancer	36	01285609
			Placebo+paclitaxel+carboplatin	361	64 (28–85)	309 (85)			
Robert et al. (19)	2011	3	Ipilimumab+dacarbazine	250	57.5	152 (60.8)	Metastatic melanoma	48	00324155
			Placebo+dacarbazine	252	56.4	149 (59.1)			

### Quality Assessment

The results of quality assessment are presented in Figure 1B. Among the 10 included studies, all studies correctly reported the methods of randomization. There was no detection bias and reporting bias among the included studies. Eight studies correctly reported allocation concealment methods, two studies showed performance bias, and one study was associated with incomplete outcome data.

### Nausea

**Figure 2:**
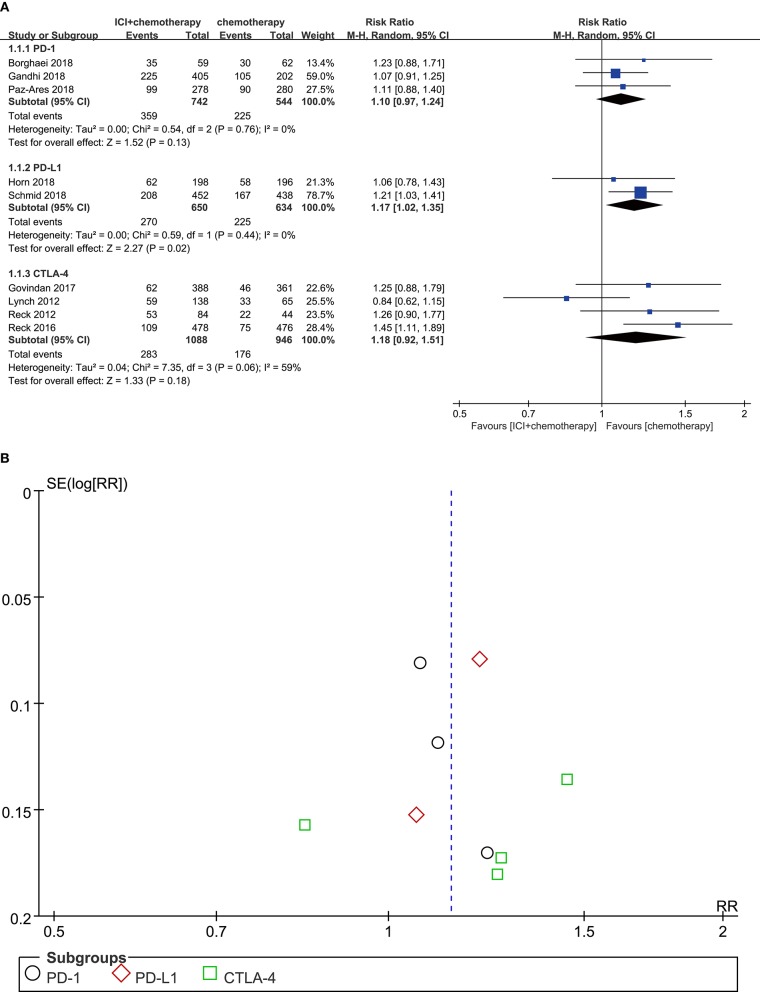
**(A)** Forest plot of nausea in patients treated with immune checkpoint inhibitor plus chemotherapy vs. chemotherapy. **(B)** Funnel plot of nausea in patients treated with immune checkpoint inhibitor plus chemotherapy vs. chemotherapy. ICI, immune checkpoint inhibitor; CTLA-4, cytotoxic T lymphocyte antigen-4; PD-L1, programmed death ligand 1; PD-1, programmed death 1; SE, standard error; RR, risk ratio.

Compared with chemotherapy alone, patients who received PD-L1 inhibitor plus chemotherapy were significantly associated with more nausea (RR = 1.17, 95% CI, 1.02–1.35, *P* = 0.020; *I*^2^ = 0%). For patients treated with PD-1 inhibitor plus chemotherapy, there was no significant difference in the risk of nausea (RR = 1.10, 95% CI, 0.97–1.24, *P* = 0.130; *I*^2^ = 0%). In addition, the difference between CTLA-4 inhibitor plus chemotherapy and chemotherapy alone was also not significant (RR = 1.18, 95% CI, 0.92–1.51, *P* = 0.180; *I*^2^ = 59%) (Figure 2A). According to the result of the funnel plot, no significant publication bias was shown in the analysis of nausea (Figure 2B).

### Vomiting

**Figure 3:**
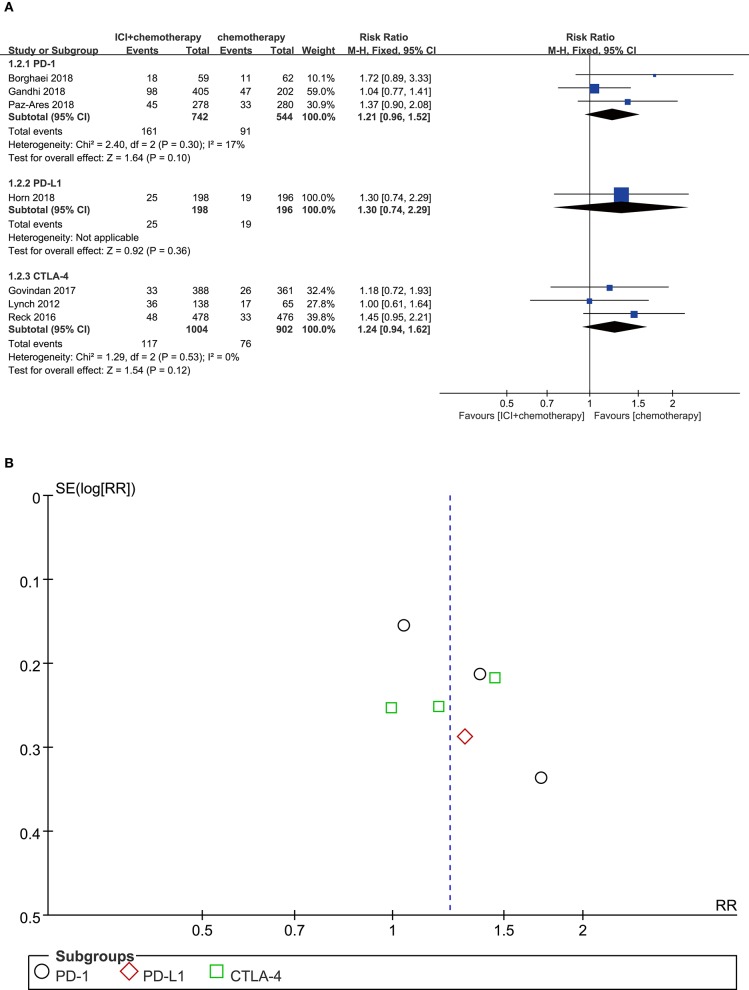
**(A)** Forest plot of vomiting in patients treated with immune checkpoint inhibitor plus chemotherapy vs. chemotherapy. **(B)** Funnel plot of vomiting in patients treated with immune checkpoint inhibitor plus chemotherapy vs. chemotherapy. ICI, immune checkpoint inhibitor; CTLA-4, cytotoxic T lymphocyte antigen-4; PD-L1, programmed death ligand 1; PD-1, programmed death 1; SE, standard error; RR, risk ratio.

Among patients treated with PD-1 inhibitor (RR = 1.21, 95% CI, 0.96–1.52, *P* = 0.100; *I*^2^ = 17%), PD-L1 inhibitor (RR = 1.30, 95% CI, 0.74–2.29, *P* = 0.360; *I*^2^ = 0%), or CTLA-4 inhibitor (RR = 1.24, 95% CI, 0.94–1.62, *P* = 0.120; *I*^2^ = 0%) plus chemotherapy, none of the comparisons showed significant increase of the vomiting risk (Figure 3A). According to the result from the funnel plot, no significant publication bias was shown in the analysis of vomiting (Figure 3B).

### Diarrhea

**Figure 4:**
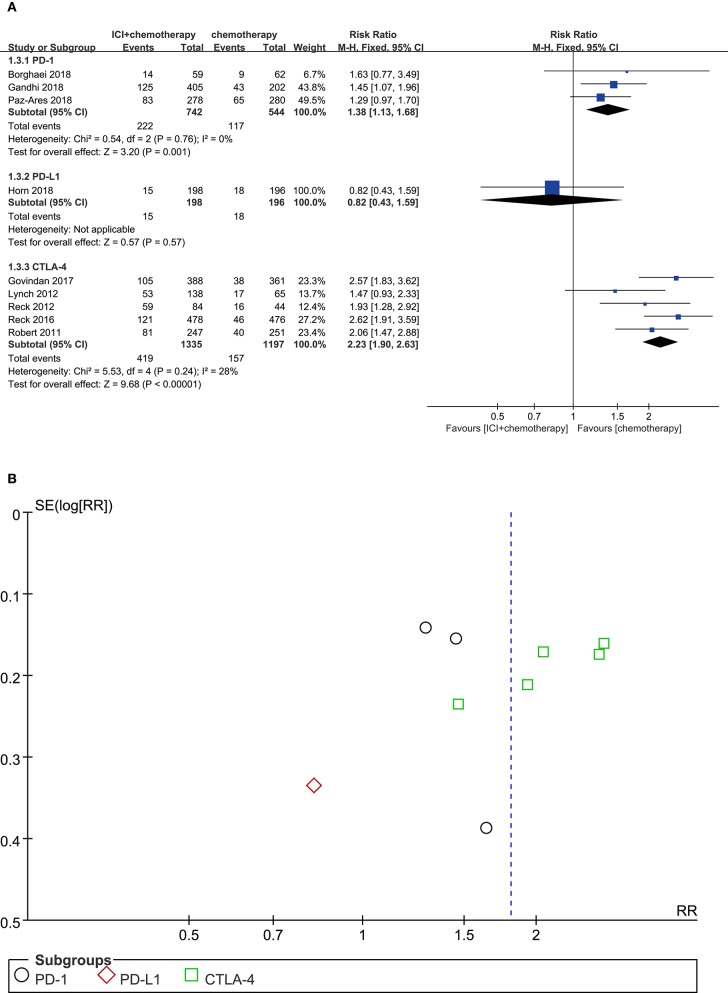
**(A)** Forest plot of diarrhea in patients treated with immune checkpoint inhibitor plus chemotherapy vs. chemotherapy. **(B)** Funnel plot of diarrhea in patients treated with immune checkpoint inhibitor plus chemotherapy vs. chemotherapy. ICI, immune checkpoint inhibitor; CTLA-4, cytotoxic T lymphocyte antigen-4; PD-L1, programmed death ligand 1; PD-1, programmed death 1; SE, standard error; RR, risk ratio.

Compared with chemotherapy alone, PD-1 inhibitor plus chemotherapy regimen was significantly associated with more diarrhea events (RR = 1.38, 95% CI, 1.13–1.68, *P* = 0.001; *I*^2^ = 0%). CTLA-4 inhibitor plus chemotherapy was also associated with a significant higher risk of diarrhea (RR = 2.23, 95% CI, 1.90–2.63, *P* < 0.00001; *I*^2^ = 0%). However, for PD-L1 inhibitor plus chemotherapy, the risk of diarrhea was not significantly increased compared with chemotherapy alone (RR = 0.82, 95% CI, 0.43–1.59, *P* = 0.570; *I*^2^ = 0%) (Figure 4A). According to the funnel plot, significant publication bias was shown in the analysis of diarrhea (Figure 4B).

### Constipation

**Figure 5:**
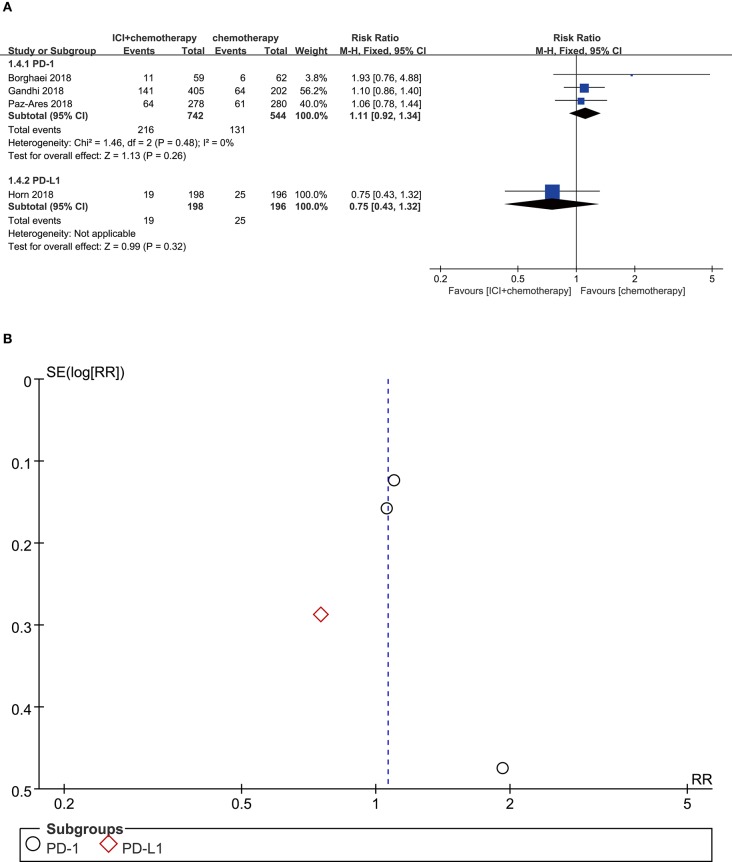
**(A)** Forest plot of constipation in patients treated with immune checkpoint inhibitor plus chemotherapy vs. chemotherapy. **(B)** Funnel plot of constipation in patients treated with immune checkpoint inhibitor plus chemotherapy vs. chemotherapy. ICI, immune checkpoint inhibitor; PD-L1, programmed death ligand 1; PD-1, programmed death 1; SE, standard error; RR, risk ratio.

Among patients treated with PD-1 inhibitor (RR = 1.11, 95% CI, 0.92–1.34, *P* = 0.260; *I*^2^ = 0%) or CTLA-4 inhibitor (RR = 0.75, 95% CI, 0.43–1.32, *P* = 0.320; *I*^2^ = 0%) plus chemotherapy, neither of the comparisons showed significant increase of the constipation risk (Figure 5A). According to the funnel plot, no significant publication bias was shown in the analysis of constipation (Figure 5B).

### Decreased Appetite

**Figure 6:**
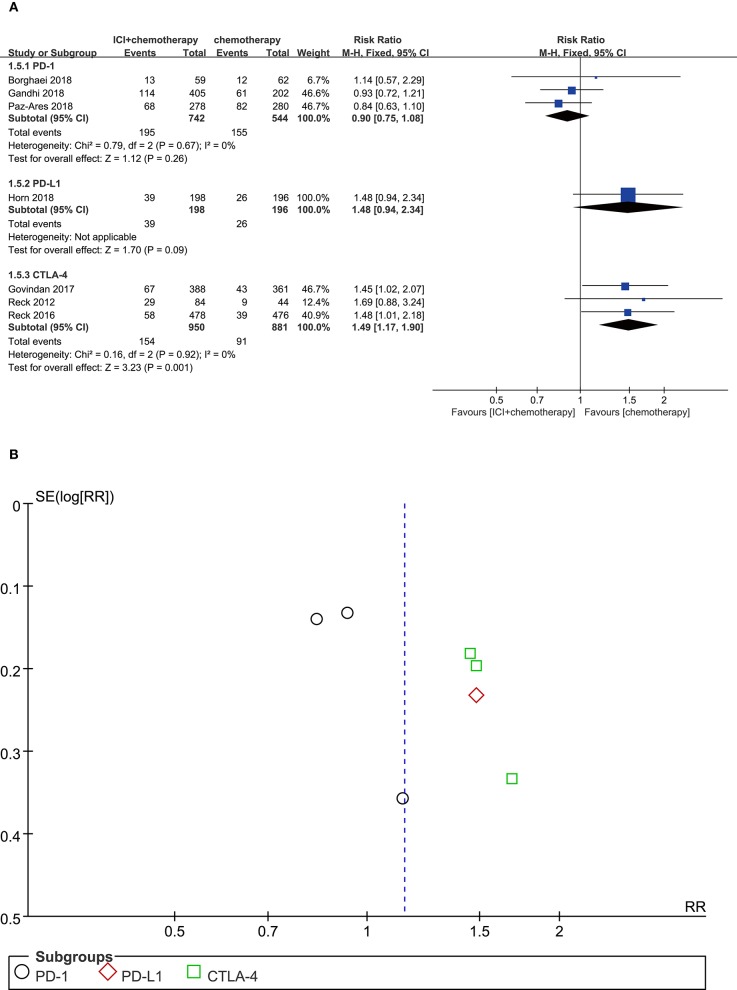
**(A)** Forest plot of decreased appetite in patients treated with immune checkpoint inhibitor plus chemotherapy vs. chemotherapy. **(B)** Funnel plot of decreased appetite in patients treated with immune checkpoint inhibitor plus chemotherapy vs. chemotherapy. ICI, immune checkpoint inhibitor; CTLA-4, cytotoxic T lymphocyte antigen-4; PD-L1, programmed death ligand 1; PD-1, programmed death 1; SE, standard error; RR, risk ratio.

Compared with chemotherapy alone, patients treated with CTLA-4 inhibitor plus chemotherapy experienced more decreased appetite events with significant differences (RR = 1.49, 95% CI, 1.17–1.90, *P* = 0.001; *I*^2^ = 0%). For patients treated with PD-1 inhibitor plus chemotherapy, the risk of decreased appetite was not significantly different compared with those who received chemotherapy alone (RR = 0.90, 95% CI, 0.75–1.08, *P* = 0.260; *I*^2^ = 0%). Furthermore, we also found that patients treated with PD-L1 inhibitor plus chemotherapy experienced no significantly more decreased appetite events compared with those with chemotherapy monotherapy (RR = 1.48, 95% CI, 0.94–2.34, *P* = 0.090; *I*^2^ = 0%) (Figure 6A). According to the funnel plot, no significant publication bias was shown in the analysis of decreased appetite (Figure 6B).

### Colitis

**Figure 7:**
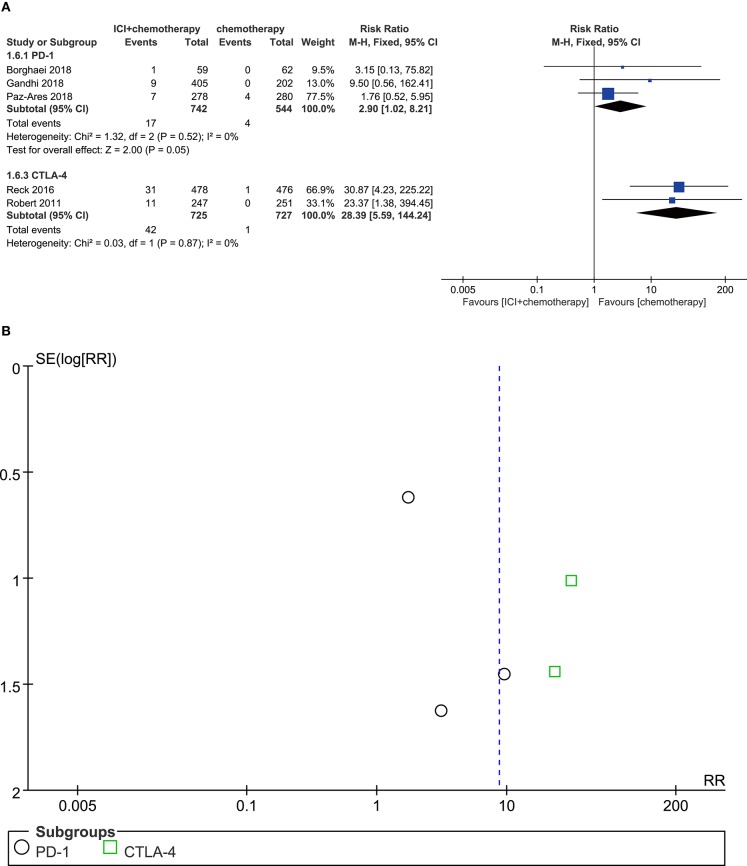
**(A)** Forest plot of colitis in patients treated with immune checkpoint inhibitor plus chemotherapy vs. chemotherapy. **(B)** Funnel plot of colitis in patients treated with immune checkpoint inhibitor plus chemotherapy vs. chemotherapy. ICI, immune checkpoint inhibitor; CTLA-4, cytotoxic T lymphocyte antigen-4; PD-1, programmed death 1; SE, standard error; RR, risk ratio.

Compared with chemotherapy alone, patients who received PD-1 inhibitor plus chemotherapy were associated with marginally significant increased risk of colitis (RR = 2.90, 95% CI, 1.02–8.21, *P* = 0.050; *I*^2^ = 0%). Moreover, patients treated with CTLA-4 inhibitors plus chemotherapy significantly developed more colitis events (RR = 28.39, 95% CI, 5.59–144.24; *P* < 0.001; *I*^2^ = 0%) (Figure 7A). According to the result from the funnel plot, no significant publication bias was shown in the analysis of colitis (Figure 7B).

## Discussion

This is the first systematic review that characterizes the risk of gastrointestinal AEs associated with the use of ICI plus chemotherapy. All trials included in this meta-analysis were basically well designed. Gastrointestinal AEs are common in both chemotherapy and ICI therapy, which deserve to be highly valued in combination. Our study demonstrated that the gastrointestinal AE profile observed was as expected on the basis of the known events, such as nausea, vomiting, diarrhea, constipation, decreased appetite, and colitis. However, compared with chemotherapy alone, a higher risk of gastrointestinal AEs was found among patients following the combination therapy of ICI plus chemotherapy.

As can be seen from the results, colitis has emerged as the most significant higher risk of gastrointestinal AEs compared to conventional chemotherapy alone. ICIs are known to have a distinct toxicity profile commonly identified as irAEs, such as pneumonia, colitis, and endocrine system disease. These irAEs are believed to be attributed to the impact of ICI on the augmentation of immunity, which may be rarely induced by conventional chemotherapy (24, 25). Most importantly, according to the subgroup analysis based on ICI types, there was a significant risk of developing colitis in patients with CTLA-4 inhibitors plus chemotherapy compared to PD-1 inhibitors plus chemotherapy. This was consistent with the previous clinical trials involving different ICI comparisons (26, 27). CTLA-4 competes with CD28 in binding to B7 to regulate cell trafficking and set the activation threshold within T cells. Because of its importance in maintenance of peripheral tolerance, CTLA-4 has been implicated in several autoimmune diseases (28). Nancey et al. showed that the blockade of CTLA-4 was related to the depleting of mucosal forkhead/winged helix transcription factor p3 (FOXP3^+^) and thus caused inflammation. PD-1 protein is another T cell co-inhibitory receptor with a structure similar to that of CTLA-4 but with a distinct biologic function and ligand specificity (29). In contrast to CTLA-4 ligands, PD-L1 is selectively expressed on tumors and cells within the tumor microenvironment (30, 31). The specific distribution may be responsible for the relatively low irAEs of the PD-1/L1 pathway inhibitor (32). It is worth emphasizing that colitis could lead to abdominal pain, perforation, or even be lethal if not promptly or properly treated (33). We also observed a significantly higher risk of diarrhea in patients receiving ICI plus chemotherapy. Specifically, diarrhea was more common among patients with combination therapy consisting of CTLA-4 inhibitor and chemotherapy. Not surprisingly, diarrhea is the most prominent symptom of colitis. As mentioned before, the blockade of CTLA-4 caused more colitis compared with that of PD-1/L1 pathway, and it was also associated with more diarrhea. Consequently, we consider it necessary for clinicians to pay close attention to the management of colitis when performing combination therapy, especially in the application of CLTA-4. Some treatment options (such as prednisone, infliximab, and vedolizumab) could be appropriate for the treatment of immune-mediated diarrhea and colitis, with favorable outcomes and good safety profiles (34).

Furthermore, nausea and vomiting were reported as the most common gastrointestinal AEs following the use of ICI plus chemotherapy (11). In the KEYNOTE-189 trial, more than half of the patients developed nausea and nearly a quarter of them developed vomiting in the pembrolizumab combination group (11). We performed a subgroup analysis of different ICI types (PD-1, PD-L1, and CTLA-4) of combination treatments and found that only PD-L1 inhibitor plus chemotherapy regimen exhibited statistically significant difference in terms of causing nausea. As for vomiting, there was no significant difference between the combination therapy and chemotherapy, regardless of the types of ICIs. Although nausea and vomiting were not considered as a life-threatening AE for cancer patients, the high incidence often resulted in the termination of treatments. The management of nausea and vomiting hence needs to be taken seriously into consideration when performing combination therapy. It might be necessary for patients to be pretreated with antiemetic agents and adrenocortical hormone agents as well. In addition, constipation was reported as a frequent AE in patients receiving chemotherapy, but rarely in ICI therapy. KEYNOTE-024 reported a 3% incidence of constipation in patients treated with pembrolizumab, while an 11.7% incidence in chemotherapy group (27). Our study demonstrated that the combination of ICI and chemotherapy did not increase the risk of constipation compared with chemotherapy alone. However, given that there were only four included studies involving constipation data, this conclusion needed to be carried out by further investigation. Finally, regarding decreased appetite, our study showed a statistically significant difference only in the CTLA-4 combination subgroup. The result of our meta-analysis showed that the regimen involving CTLA-4 inhibitor led to higher risk of gastrointestinal AEs compared with other ICIs, which prompted oncologists to pay more attention to the management of gastrointestinal AEs in patients receiving CTLA-4 inhibitor and its combination regimens (35).

Studies have shown that the mucosal immune system (MIS) not only is the first defense barrier against oral pathogens but also constitutes an important part of the body's entire immune network (36). ICIs exert anti-tumor effects by blocking the inhibitory receptors of immunity. At the same time, it may also disturb immunologic homeostasis and thus cause various irAEs. In previous clinical studies, gastrointestinal irAEs, such as colitis and diarrhea, were commonly reported among patients receiving ICI monotherapy. In a meta-analysis, the incidence of all grade colitis was estimated to be 8.8% and 1.6% in CTLA-4 inhibitor and PD-1 inhibitor treatment groups, respectively (37). On the other hand, chemotherapy drugs cause intestinal dysbacteriosis or directly kill intestinal mucosal cells, both of which may mediate the overexpression of inflammatory cytokines and result in corresponding gastrointestinal side effects. As both types of drugs are associated with the injury of the gastrointestinal mucosa, it may be reasonable that more gastrointestinal toxicities occur when immunotherapy is combined with chemotherapy.

Although this study has answered some important questions, there are still several limitations we should state. Firstly, this study included different types of gastrointestinal adverse effects with an incidence of more than 15% other than immune-related AEs, while it did not include the adverse effect with a lower incidence such as gastrointestinal hemorrhage. Therefore, this might lead to the overestimation of the safety of ICI plus chemotherapy. In addition, significant publication bias was shown in the analysis of diarrhea, which might lead to unreliable and misleading information in this meta-analysis. Furthermore, whether the increased risk of gastrointestinal AEs was due to the addition of ICI or the possible synergy mechanisms between two treatments (ICI plus chemotherapy) remains unknown, which needs further investigation. Finally, data on the gastrointestinal event leading to discontinuation of treatment, which is also an important gastrointestinal safety outcome, was not reported. Further studies are warranted to analyze this issue.

In conclusion, this meta-analysis demonstrates that ICI plus chemotherapy regimen is associated with a higher risk of gastrointestinal AEs such as nausea, diarrhea, vomiting, and colitis compared with chemotherapy alone, while the risk of constipation and decreased appetite were not increased. Moreover, combining different ICIs (PD-1, PD-L1, or CTLA-4) might lead to different gastrointestinal toxicity. These increased gastrointestinal AEs may restrict the applications of ICI plus chemotherapy regimen, and the management is of great importance for clinicians.

## Author Contributions

WY and PM contributed to study selection, data extraction, quality assessment, statistical analysis and drafting of the manuscript. HX contributed to data acquisition, data interpretation. MJ and QL contributed to the study design and revision of the manuscript. All the authors have final approval of the submitted manuscript and reached agreement to be accountable for all aspects of the work.

### Conflict of Interest

The authors declare that the research was conducted in the absence of any commercial or financial relationships that could be construed as a potential conflict of interest.
